# Supplementary material for: Comparative genomics reveals the origin of fungal hyphae and multicellularity
Source: Nat Commun. 2019 Sep 9;10:4080. doi: 10.1038/s41467-019-12085-w (PMC6733946; doi:10.1038/s41467-019-12085-w)
Supplement: Supplementary file 1 — Supplementary Information [file 41467_2019_12085_MOESM1_ESM.pdf]

Supplementary Information

Comparative genomics reveals the origin of fungal hyphae and multicellularity

Kiss et al.

Contents:

Supplementary Note 1. Copy number analysis of Fonticula alba and its relationship to fungal multicellularity.
Supplementary Figure 1. Phylogenetic trees inferred using RAxML, IQ-tree and PhyloBayes.
Supplementary Figure 2. Reconstructed gene duplication/loss histories across all the gene families in the examined genomes
Supplementary Figure 3. Reconstructions of ancestral gene copy numbers of histidine-kinase genes.
Supplementary Figure 4. Conservation of hypha morphogenesis genes across fungi.
Supplementary Figure 5. Phylogenetic age distribution of hypha morphogenesis genes.
Supplementary Figure 6. Statistical comparisons of basic structural properties of genes related to hyphal MC.
Supplementary Figure 7. Comparison of the dynamics of PFAM domain evolution between plesiomorphically unicellular and multicellular fungi.
Supplementary Figure 8. Reconstructions of ancestral gene copy numbers of NADPH-oxidase genes.
Supplementary References.

Supplementary Note 1

A complete genome of *Fonticula alba*, a representative of the sister group of Fungi became available recently. *Fonticula* forms simple multicellular fruiting bodies which arise by the aggregation of individual cells (termed cellular slime mold habit[1–5]). As such, its multicellularity differs from hyphal multicellularity in fungi, but it is nevertheless interesting if any of the genes we examined in fungi also show expansions/contractions in *Fonticula*. The genome of *Fonticula alba* became available after the completion of our analyses, but considering its importance – its sister position to fungi and aggregative multicellular lifestyle – we complemented our analysis with its genome and performed copy number-based analyses using the same methods as for fungi.

First we investigated Ser/Thr and histidine kinases, G-protein coupled receptors (GPCRs) and adhesive proteins. We found that *Fonticula* has much fewer Ser/Thr kinases (323) than metazoans (mean 644) and it was more similar to gene copy numbers of non-fungal opisthokonts (mean 392) than to fungi (mean 260). Considerably fewer histidine kinases were found in *Fonticula* (10) than in multicellular fungi (mean 23)(Suppl. Fig. 3). We detected no GPCRs in *Fonticula* (Fig. 2b). The adhesion gene set (6), in turn, was similar to that of protists, early fungi and the Mucoromycotina, although it should be noted that all these groups have poor adhesion gene repertoires.

We also focused on the main hyphal multicellularity-related functional categories and analyzed their copy numbers in *Fonticula alba* compared to the other 71 species (Suppl. Data 2). In "cell wall biogenesis" and "transcriptional regulation" categories, where we found significant gene family expansions potentially related to hyphal multicellularity, *Fonticula* had low gene copy numbers, resembling protists and animals rather than fungal lineages. In terms of "polarity maintenance" genes, *Fonticula* was most similar to other non-fungal eukaryotes like *Monosiga* and *Capsaspora*, and to plesiomorphically unicellular fungi (*Rozella*, *Catenaria*, *Batrachochytrium* species). On the other hand, in gene groups that were more diverse in non-fungal lineages (e.g. vesicle transport, actin cytoskeleton), *Fonticula* showed transitional copy numbers between non-fungal protists and fungi. For example in "vesicle transport" genes, *Fonticula* showed the same pattern as fungi compared to non-fungal species. In "actin cytoskeleton" genes we observed a reduction of gene repertoire in *Fonticula* (27) compared to fungi (mean 34), metazoa (mean 47) and protists (mean 45) Microtubule transport-related genes were generally more diverse in Metazoa (mean 101), protists (mean 51), early fungi (mean 70), Mucoromycota (mean 56) and Agaricomycotina (mean 77), than in *Fonticula* (25). The genetic toolkit for signaling was small in *Fonticula* (52) compared to animals (mean 148), protists (mean 85) and most of fungi (mean 77). Phagocytosis-related genes were conserved in *Fonticula alba,* including two families (WAVE and WASH) that were lost in fungi, indicating an overall similarity to non-fungal protists rather than fungi (Fig. 4.).

Taken together, these analyses reveal that in the examined multicellularity-related genes *Fonticula* resembles the non-fungal protists more than it does fungi, which accords well with the independent origin of a different type of multicellularity (aggregative) in *Fonticula*[2].

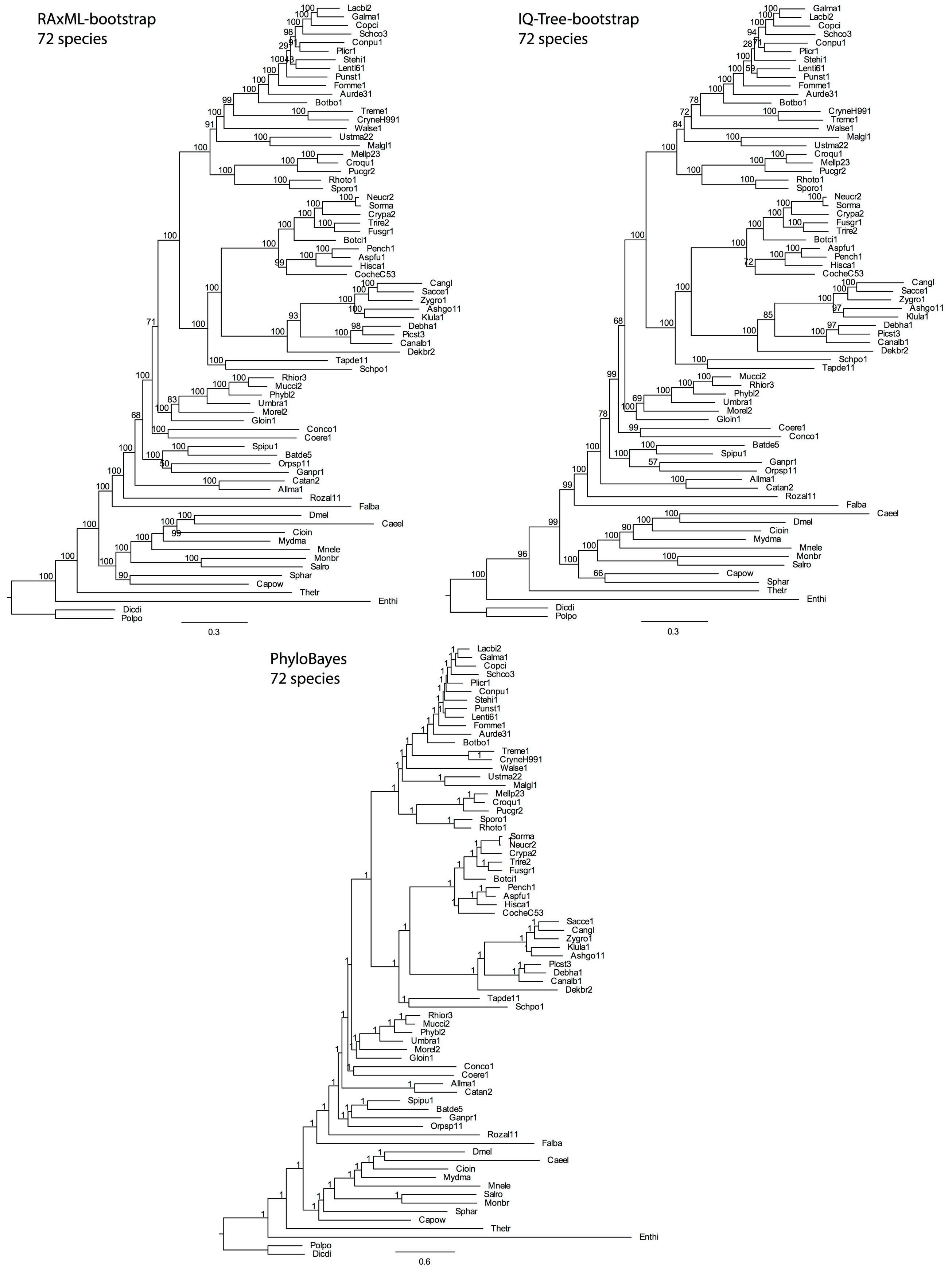

Supplementary Fig. 1. Phylogenetic trees inferred from 455 single-copy orthologs using three different methods.

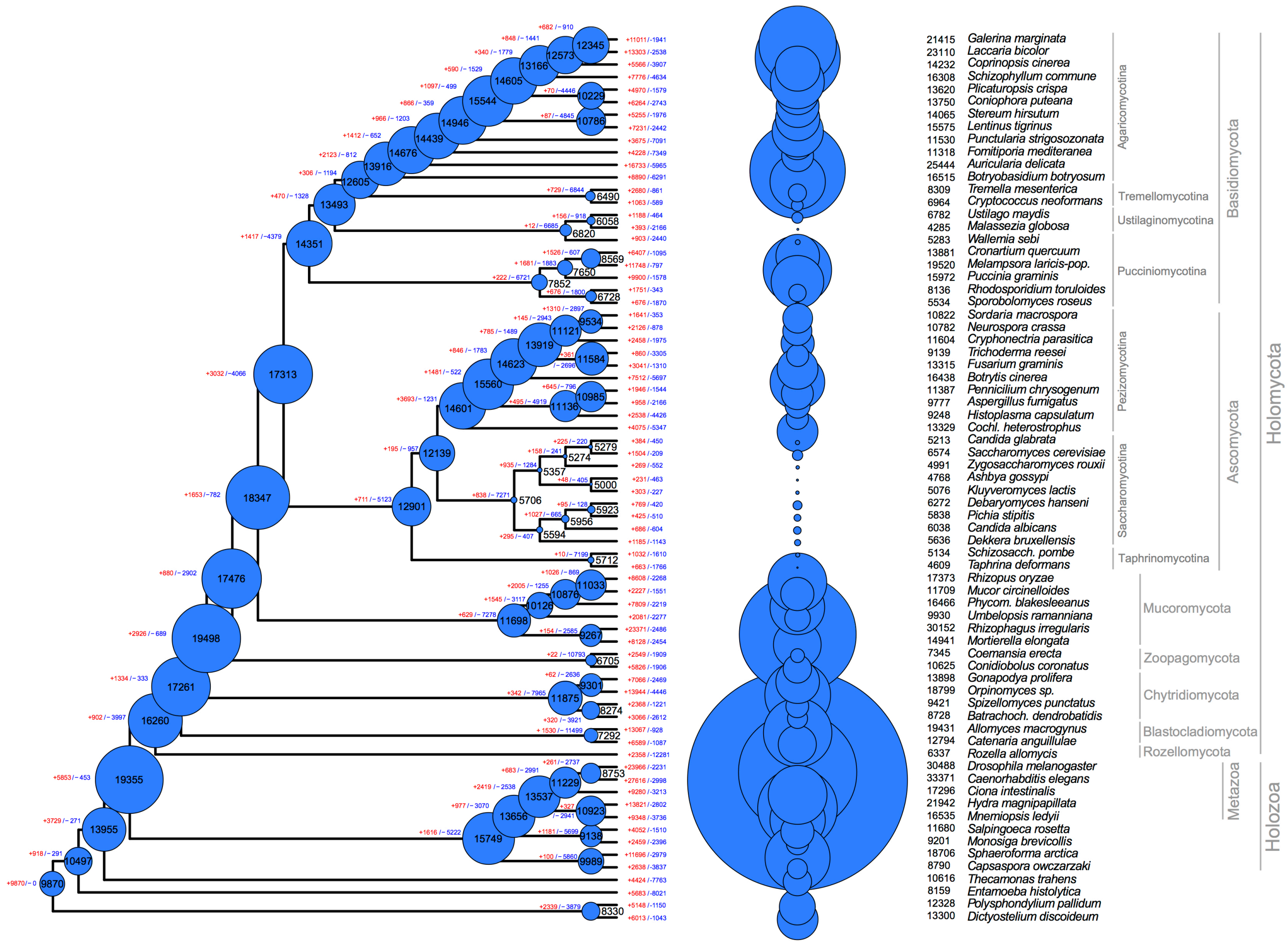

Supplementary Figure 2. Reconstructed ancestral genome sizes and gene duplication/loss histories across the examined genomes. Red and blue numbers represent gene duplications and losses across the tree. Numbers at right side represent extant gene copy numbers per species.

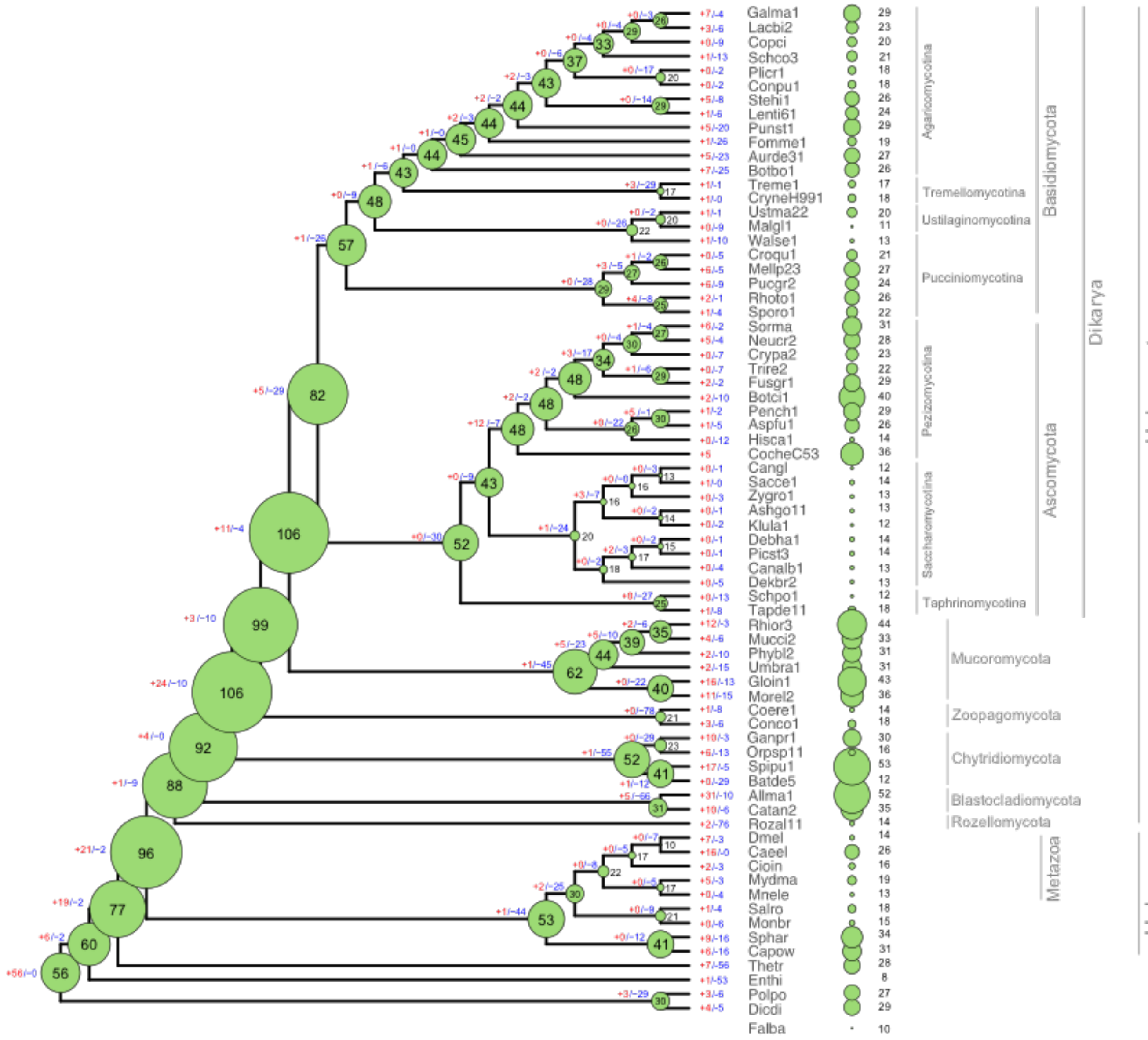

Supplementary Figure 3. Reconstructions of ancestral gene copy numbers of histidine-kinase genes. Numbers at the branches represent gene duplications (+) and losses (-) inferred by COMPARE. Bubble size is proportional to reconstructed ancestral gene copy number. Copy number distribution for each species is shown right to the tree.

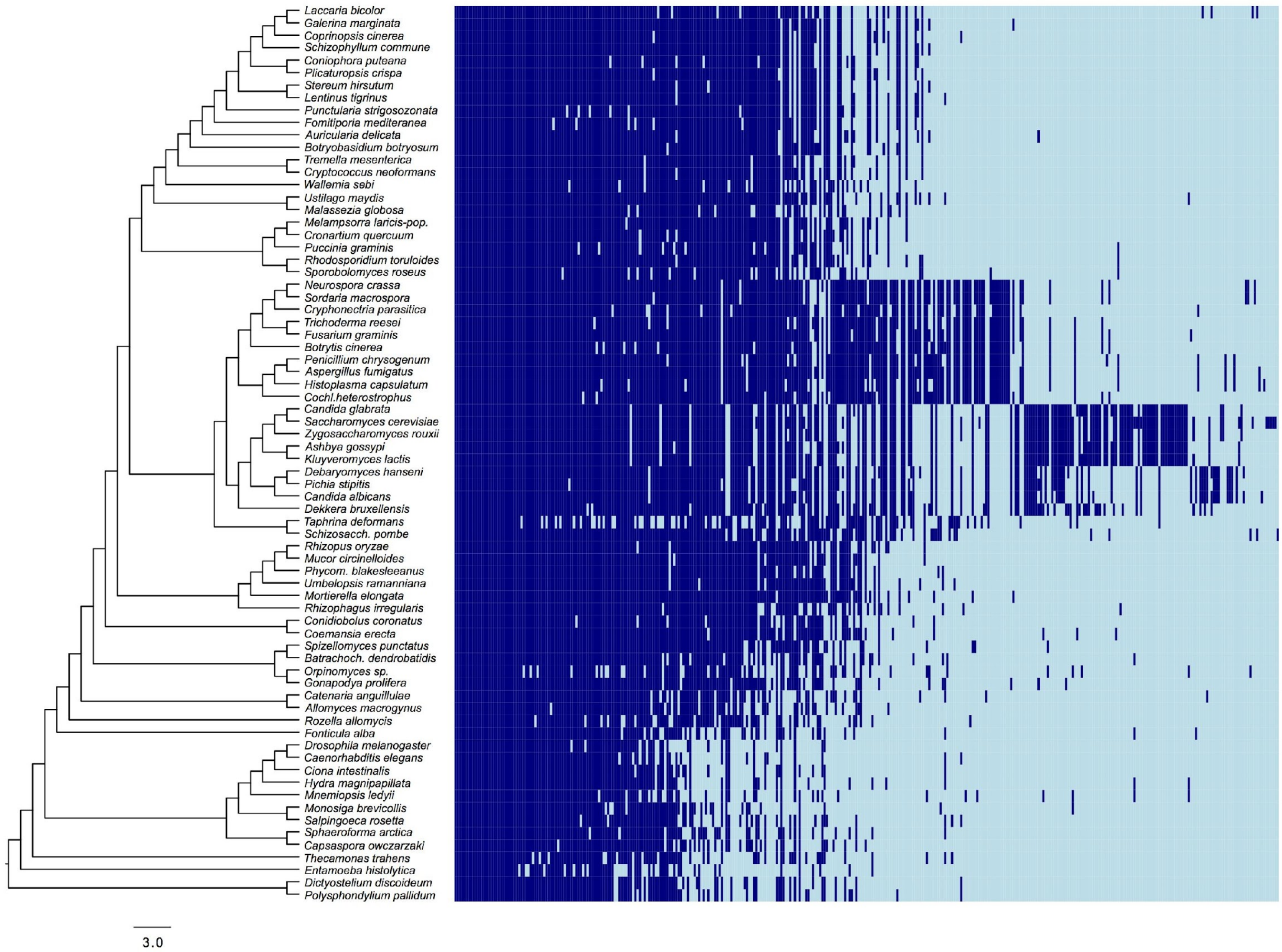

Supplementary Fig. 4. The conservation of hypha morphogenesis genes across fungi. Dark and light blue indicates that the given gene family is present and absent in a species' genome, respectively.

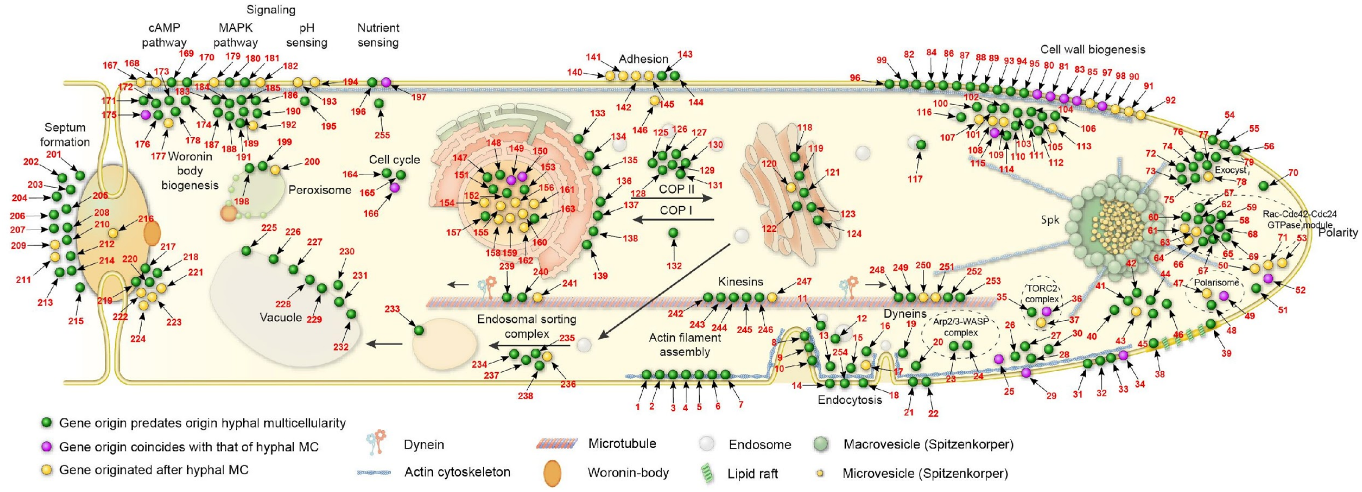

1 pfy1_Afu4g03050
2 act1_Afu6g04740
3 Afu5g10570 (cofilin in A.nidulans)
4 Afu1g03060
5 Afu3g09350
6 Afu6g10060
7 Afu2g14270
8 Afu3g12080 (arfB in A.nidulans)
9 Afu4g10020
10 Afu5g07930
11 Afu4g08040 (rabB in A.nidulans)
12 Afu2g02610
13 Afu2g01200
14 Afu6g02070 (dnfA in A.nidulans)
15 Afu7g03680
16 sac6_Afu2g07420
17 Afu2g10320
18 Afu2g08850 (dnfB in A.nidulans)
19 Afu8g02850
20 Afu6g02540
21 Afu5g05950
22 Afu3g14230
23 Afu1g13330 (arp2)
24 Afu8g05660 (myoA in A.nidulans)
25 Afu7g03870
26 Afu1g05930
27 pil1_Afu6g07520
28 Afu3g06140 (slaB in A.nidulans)
29 Afu8g04520
30 Afu1g13020
31 Afu4g03160
32 Afu5g05710 (swoC in A.nidulans)
33 Afu1g02900
34 rgsB_Afu4g12640
35 Afu2g10460
36 Afu6g07910
37 sin1_Afu2g12210
38 Afu3g13440 (stoA in A.nidulans)
39 Afu4g06290 (barA in A.nidulans)
40 stt4_Afu7g03760
41 Afu3g06080
42 Afu2g02530
43 Afu2g08180 (floA in A.nidulans)
44 erg9_Afu7g01220
45 Afu4g08070 (swoF in A.nidulans)
46 Afu6g12920
47 Afu1g09640
48 Afu2g03710 (spaA in A.nidulans)
49 Afu6g04940 (sepA in A.nidulans)
50 Afu1g11920 (teaC in A.nidulans)
51 Afu1g02920 (mesA in A.nidulans)
52 Afu4g04120
53 Afu1g06090 (teaR in A.nidulans)
54 csmB_Afu2g13430
55 chsE_Afu2g13440
56 fks1_Afu6g12400
57 Afu8g02810 (elmo_like in A.nidulans)
58 Afu2g05740 (modA in A.nidulans)
59 racA_Afu3g06300
60 rho1_Afu6g06900
61 Afu1g04340 (dock180_like in A.nidulans)
62 Afu2g01060
63 Afu3g06280
64 Afu5g07430
65 rho4_Afu5g14060
66 Afu2g15050
67 Afu4g11450 (cdc24 in A.nidulans)
68 Afu5g11380
69 rom2_Afu5g08550
70 rasA_Afu5g11230
71 Afu2g02520 (teaA in A.nidulans)
72 Afu6g11370 (exo84 in A.nidulans)
73 Afu1g12790 (sec5 in A.nidulans)
74 Afu8g02790
75 Afu2g11960 (exo70 in A.nidulans)
76 Afu6g05290 (sec15 in A.nidulans)
77 Afu4g10490 (sec6 in A.nidulans)
78 Afu1g04360 (secC in A.nidulans)
79 Afu5g08040 (secH in A.nidulans)
80 gel7_Afu6g12410
81 crh3_Afu3g09250
82 ecm33_Afu4g06820
83 kre6_Afu2g11870
84 chiA1_Afu5g03760
85 Afu4g00620
86 chsA_Afu2g01870
87 chsB_Afu4g04180
88 chsC_Afu5g00760
89 chsG_Afu3g14420
90 scw4_Afu6g12380
91 cfmA_Afu6g14090
92 ags1_Afu3g00910
93 exg1_Afu1g03600
94 chs3_Afu8g05620
95 bgt1_Afu1g11460
96 neg1_Afu8g07120
97 sun1_Afu7g05450
98 chsD_Afu1g12600
99 chsF_Afu8g05630
100 orlA_Afu3g05650
101 pld3_Afu3g05630
102 Afu5g05550 (myoV_myoE in A.nidulans)
103 ddiA_Afu7g06050
104 Afu7g01510
105 Afu6g02920 (synA in A.nidulans)
106 Afu5g10810
107 Afu2g07590
108 Afu5g05770
109 Afu3g05580
110 Afu2g13760
111 Afu6g02510
112 Afu1g12040
113 ags2_Afu2g11270
114 Afu3g12800
115 Afu8g02320
116 gfa1_Afu6g06340
117 Afu5g08670
118 Afu1g10970
119 Afu6g04840 (hypA in A.nidulans)
120 Afu5g03580 (podB in A.nidulans)
121 Afu4g10040
122 kexB_Afu4g12970
123 Afu5g11900
124 Afu4g08050
125 Afu6g03840
126 Afu6g07290
127 Afu1g11470
128 Afu3g08840 (copA in A.nidulans)
129 Afu4g07390
130 Afu3g09700
131 Afu4g13190
132 hypB_Afu7g05700
133 pmt2_Afu1g07690
134 pmt4_Afu8g04500
135 Afu2g12580
136 Afu2g02360
137 Afu5g13210
138 cwh41_Afu6g04210
139 Afu1g05470
140 rodA_Afu5g09580
141 rodB_Afu1g17250
142 rodE_Afu8g05890
143 glfB_Afu3g12700
144 Afu2g12180
145 Afu1g01540
146 gprA_Afu3g02970
147 Afu8g05570
148 hsf1_Afu5g01900
149 stuA_Afu2g07900
150 medA_Afu2g13260
151 sebA_Afu4g09080
152 MAT1-2_Afu3g06170
153 Afu3g09640
154 Afu2g05020 (dopA in A.nidulans)
155 Afu4g04502
156 Afu2g10770
157 steA_Afu5g06190
158 Afu2g12910
159 Afu7g02260
160 ace2_Afu3g11250
161 pacC_Afu3g11970
162 wetA_Afu4g13230
163 Afu2g16840
164 Afu4g12160
165 Afu8g04810
166 Afu4g06130
167 gprD_Afu2g12640
168 gprC_Afu7g04800
169 gprI_Afu3g00780
170 gprL_Afu3g01750
171 sfaD_Afu5g12210
172 gpaA_Afu1g13140
173 gpaB_Afu1g12930
174 Afu1g13250
175 ricA_Afu4g08820
176 acyA_Afu6g08520
177 pdeB_Afu1g05230
178 pkaR_Afu3g10000
179 preA_Afu5g07880
180 sho1_Afu5g08420
181 Afu3g09740
182 preB_Afu3g14330
183 ssk1_Afu5g08390
184 sskB_Afu1g10940
185 ptcG_Afu5g13340
186 Afu5g06420
187 sakA_Afu1g12940
188 mpkB_Afu6g12820
189 artA_Afu2g03290
190 sitA_Afu6g11470
191 ypd1_Afu4g10280
192 Afu4g04070
193 Afu2g14900
194 palH_Afu5g13270
195 Afu4g06400
196 meaA_Afu2g05880
197 Afu2g08800
198 pex11_Afu6g07740
199 Afu2g17080 (wscA in A.nidulans)
200 hexA_Afu5g08830
201 Afu6g02840
202 aspC_Afu5g03080
203 Afu1g02940
204 aspB_Afu7g05370
205 Afu6g10330
206 aspA_Afu5g08540
207 aspE_Afu3g07015
208 Afu5g02220
209 Afu1g13170
210 Afu2g12390 (mobA in A.nidulans)
211 Afu8g05680
212 Afu6g11010
213 cdcA_Afu3g12250
214 Afu5g08700 (myoB in A.nidulans)
215 hymA_Afu3g12480
216 Afu6g06520
217 Afu7g05950
218 Afu5g03940
219 calA_Afu5g09360
220 cnaB_Afu6g04540
221 Afu5g03400
222 ENGL1_Afu1g04260
223 bud4_Afu2g08470
224 Afu5g11890 (bud3 in A.nidulans)
225 Afu1g04080 (vps52 in A.nidulans)
226 Afu4g07110
227 Afu5g13540
228 Afu1g05280 (vps8 in A.nidulans)
229 Afu6g04870 (vps45 in A.nidulans)
230 Afu5g02360 (vpsA in A.nidulans)
231 Afu1g10810
232 Afu2g13750 (hbrA in A.nidulans)
233 Afu2g04740 (vps27 in A.nidulans)
234 Afu1g09220 (vps20 in A.nidulans)
235 Afu5g13890 (vps24 in A.nidulans)
236 Afu3g14380 (vps23 in A.nidulans)
237 Afu1g06420 (vps32 in A.nidulans)
238 Afu4g04100
239 Afu1g13390 (mipA in A.nidulans)
240 eb1_Afu3g11860
241 Afu2g08190 (coA in A.nidulans)
242 Afu2g03150
243 Afu7g01400
244 Afu8g04660 (clipA in A.nidulans)
245 Afu6g14220
246 Afu2g14730
247 Afu7g03710 (KipA in A.nidulans)
248 Afu3g10720
249 Afu2g13450 (nudM in A.nidulans)
250 Afu5g08430
251 Afu5g08510
252 Afu6g12970
253 Afu5g11810 (nudA in A.nidulans)
254 Afu6g12570
255 Afu3g10530

Supplementary Figure 5. Phylogenetic age distribution of hypha morphogenesis genes. Figure mirrors main text Figure 3 with gene names provided for each dot.

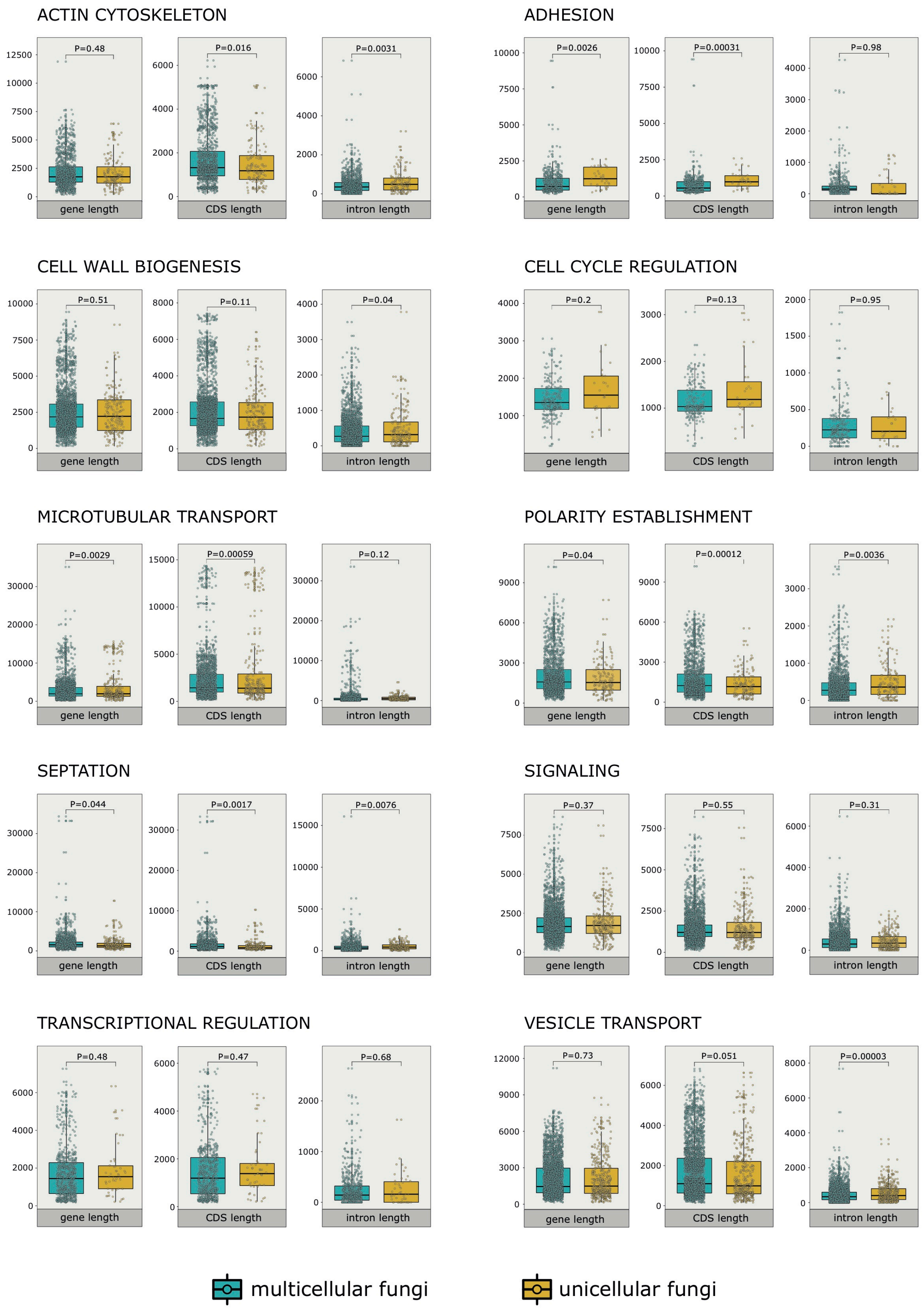

Supplementary Figure 6. Statistical comparisons of basic structural properties of genes related to hyphal MC. Box plots display differences in gene („gene length”), coding sequence („CDS

length") and intron lengths of 4 unicellular (yellow) and 39 multicellular fungi (blue) in nine functional categories. P-values derived from independent two-tailed Welch's t-tests are given for each comparison. Vertical lines on boxplots represent lower and upper quartiles.

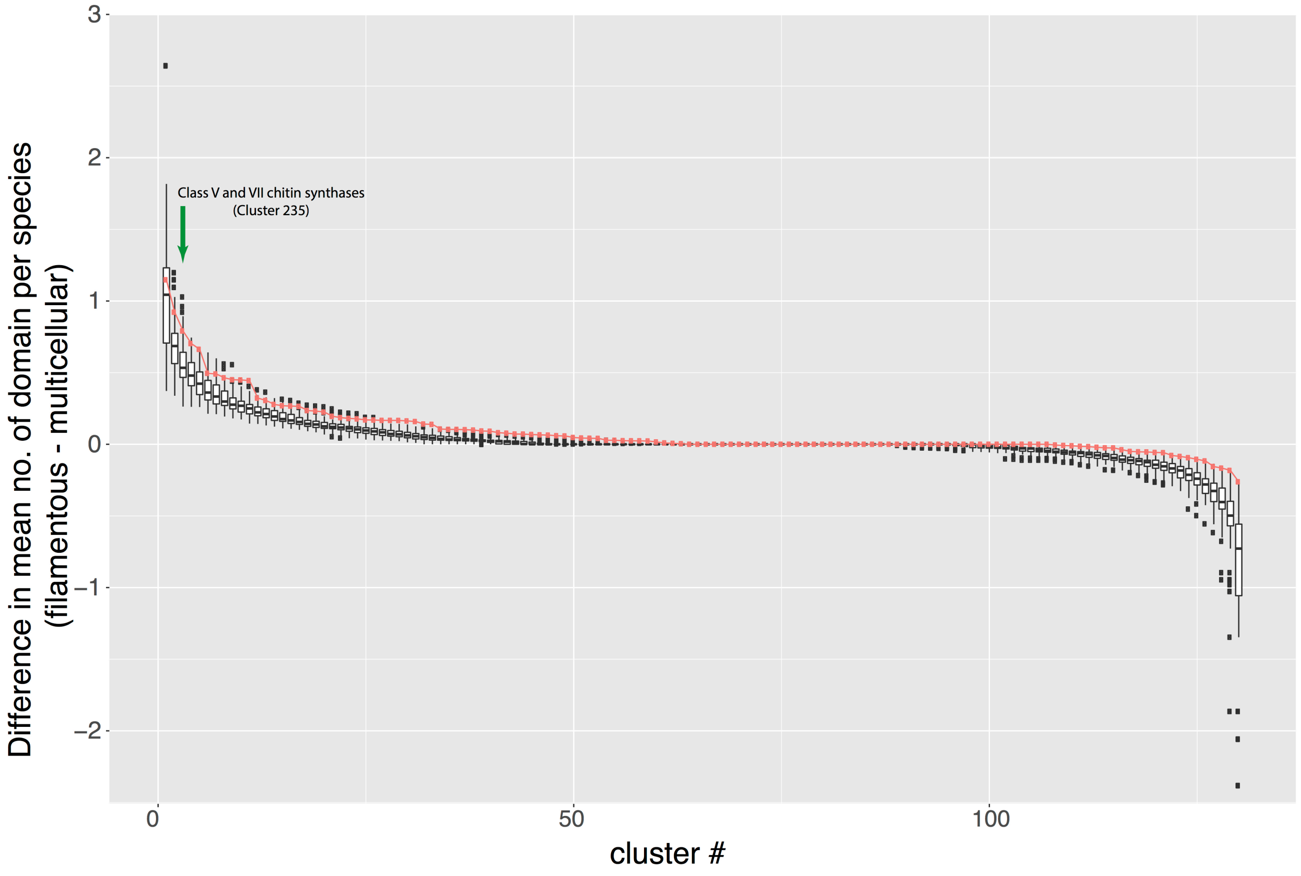

Supplementary Figure 7. Comparison of the dynamics of PFAM domain evolution between plesiomorphically unicellular and multicellular fungi. The plot shows for each of the morphogenesis-related gene families the difference between the mean number of domains per protein in unicellular and filamentous fungi (red dots). Boxplots (and corresponding outlier black dots) indicate the distribution of the same values across 100 randomly chosen clusters from the 72 genomes. Positive values indicate clusters in which proteins of multicellular fungi have, on average, more pfam domains, than unicellular fungi. Only clusters in which >=70% of both unicellular and multicellular fungi are represented were considered in this analysis (129 out of 362 hypha morphogenesis-related clusters). Vertical lines on boxplots represent lower and upper quartiles.

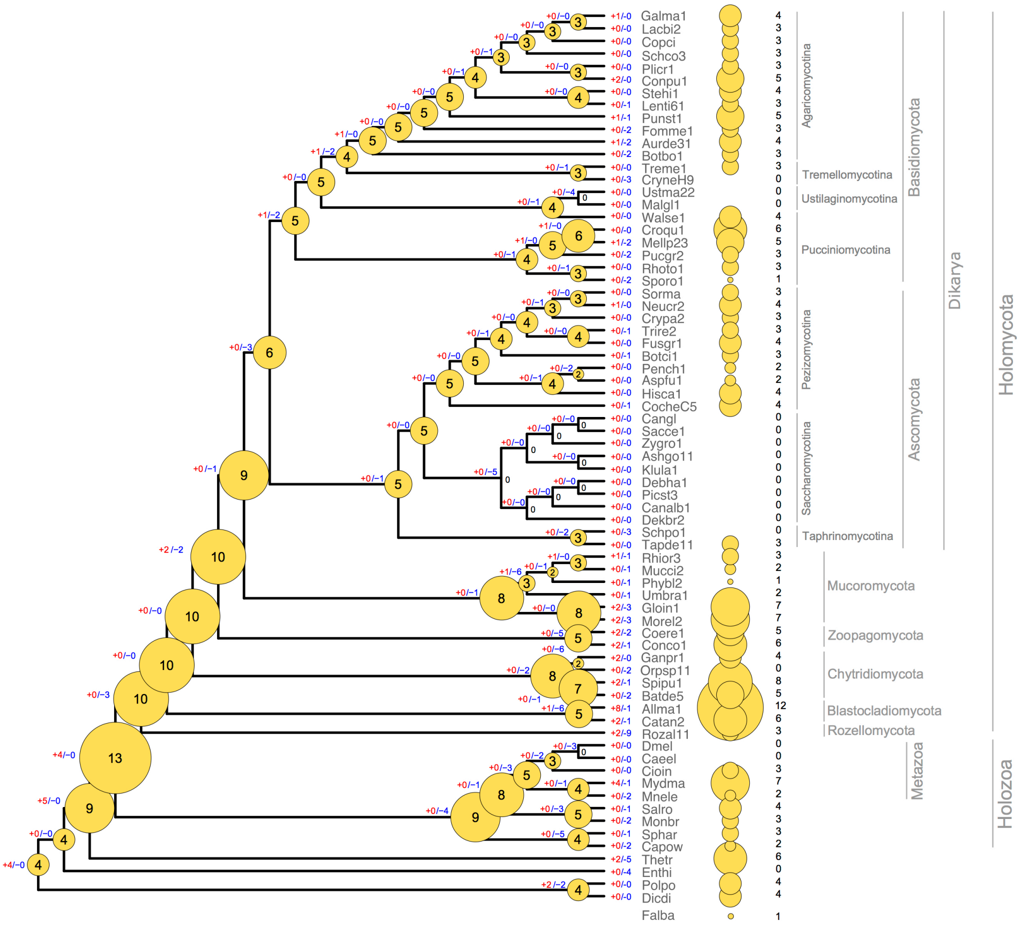

Supplementary Fig. 8. Reconstructions of ancestral gene copy numbers of NADPH-oxidase genes. Numbers at the branches represent gene duplications (+) and losses (-) inferred by COMPARE. Bubble size is proportional to reconstructed ancestral gene copy number. Copy number distribution for each species is shown right to the tree.

Supplementary references

1. Brown, M. W., Spiegel, F. W. & Silberman, J. D. Phylogeny of the Cellular Slime Mold, Fonticula alba, Reveals a Key Evolutionary Branch within Opisthokonta. *Mol. Biol. Evol.* **26**, 2699–2709 (2009).
2. Brown, M. W., Kolisko, M., Silberman, J. D. & Roger, A. J. Aggregative Multicellularity Evolved Independently in the Eukaryotic Supergroup Rhizaria. *Curr. Biol.* **22**, 1123–1127
3. Paps, J. & Ruiz-Trillo, I. Animals and Their Unicellular Ancestors. in *Encyclopedia of Life Sciences* (John Wiley & Sons, Ltd, 2010). doi:10.1002/9780470015902.a0022853
4. Paps, J., Medina-Chacón, L. A., Marshall, W., Suga, H. & Ruiz-Trillo, I. Molecular Phylogeny of Unikonts: New Insights into the Position of Apusomonads and Ancyromonads and the Internal Relationships of Opisthokonts. *Protist* **164**, 2–12 (2013).
5. Torruella, G. *et al.* Phylogenomics Reveals Convergent Evolution of Lifestyles in Close Relatives of Animals and Fungi. *Curr. Biol.* **25**, 2404–2410 (2015).
