# Supplementary material for: Comparative genomics reveals the origin of fungal hyphae and multicellularity
Source: Nat Commun. 2019 Sep 9;10:4080. doi: 10.1038/s41467-019-12085-w (PMC6733946; doi:10.1038/s41467-019-12085-w)
Supplement: Supplementary file 3 — Reporting Summary [file 41467_2019_12085_MOESM3_ESM.pdf]

nature research

Corresponding author(s): Laszlo G. Nagy

Last updated by author(s): Aug 5, 2019

Reporting Summary

Nature Research wishes to improve the reproducibility of the work that we publish. This form provides structure for consistency and transparency in reporting. For further information on Nature Research policies, see Authors & Referees and the Editorial Policy Checklist .

Statistics

For all statistical analyses, confirm that the following items are present in the figure legend, table legend, main text, or Methods section.

n/a	Confirmed	
☐	☒	The exact sample size (*n*) for each experimental group/condition, given as a discrete number and unit of measurement
☒	☐	A statement on whether measurements were taken from distinct samples or whether the same sample was measured repeatedly
☐	☒	The statistical test(s) used AND whether they are one- or two-sided<br>*Only common tests should be described solely by name; describe more complex techniques in the Methods section.*
☒	☐	A description of all covariates tested
☐	☒	A description of any assumptions or corrections, such as tests of normality and adjustment for multiple comparisons
☐	☒	A full description of the statistical parameters including central tendency (e.g. means) or other basic estimates (e.g. regression coefficient) AND variation (e.g. standard deviation) or associated estimates of uncertainty (e.g. confidence intervals)
☐	☒	For null hypothesis testing, the test statistic (e.g. *F*, *t*, *r*) with confidence intervals, effect sizes, degrees of freedom and *P* value noted<br>*Give P values as exact values whenever suitable.*
☐	☒	For Bayesian analysis, information on the choice of priors and Markov chain Monte Carlo settings
☐	☒	For hierarchical and complex designs, identification of the appropriate level for tests and full reporting of outcomes
☒	☐	Estimates of effect sizes (e.g. Cohen's *d*, Pearson's *r*), indicating how they were calculated

Our web collection on statistics for biologists contains articles on many of the points above.

Software and code

Policy information about availability of computer code

Data collection	Our study is based on published genome sequences downloaded from public repositories.
Data analysis	mpiBLAST 1.6.0111, PRANK release 140603, MAFFT version 7.222, trimAl 1.4.rev15, FastTree 2.1, RAxML 8.2.4118, Phytools v0.6-60124, IQ-TREE v1.6.10, Phylobayes 3

For manuscripts utilizing custom algorithms or software that are central to the research but not yet described in published literature, software must be made available to editors/reviewers. We strongly encourage code deposition in a community repository (e.g. GitHub). See the Nature Research guidelines for submitting code & software for further information.

Data

Policy information about availability of data

All manuscripts must include a data availability statement. This statement should provide the following information, where applicable:

- Accession codes, unique identifiers, or web links for publicly available datasets
- A list of figures that have associated raw data
- A description of any restrictions on data availability

Files associated with this paper (species trees, gene duplication/loss catalogs, concatenated alignments) have been deposited as Supplementary Data associated with the paper. Extra data (including 776 individual gene trees used in the COMPARE analysis) are available from the corresponding author upon requests.

Field-specific reporting

Please select the one below that is the best fit for your research. If you are not sure, read the appropriate sections before making your selection.

Ecological, evolutionary & environmental sciences study design

All studies must disclose on these points even when the disclosure is negative.

Study description	Our study is a comparative genomic analysis of 72 whole genomes.
Research sample	n.a.
Sampling strategy	Genomes were sampled so as to accurately represent the diversity of cellularity levels in fungi.
Data collection	Via downloading from public databases.
Timing and spatial scale	n.a.
Data exclusions	Microsporidia was excluded from phylogenetic and genomic analyses because of the excessive rate of their molceular evolution.
Reproducibility	All findings can be reproduced from the deposited data and the published methods. Whenever relevant, at least three replicates of analyses were performed to ensure the reproducibility. In all cases the replicated analyses were in concordance with each other.
Randomization	n.a.
Blinding	We performed comparative phylogenetic analyses where blinding was not relevant.

Did the study involve field work? ☐ Yes ☒ No

Reporting for specific materials, systems and methods

We require information from authors about some types of materials, experimental systems and methods used in many studies. Here, indicate whether each material, system or method listed is relevant to your study. If you are not sure if a list item applies to your research, read the appropriate section before selecting a response.

Materials & experimental systems

n/a	Involved in the study
☒	☐ Antibodies
☒	☐ Eukaryotic cell lines
☒	☐ Palaeontology
☒	☐ Animals and other organisms
☒	☐ Human research participants
☒	☐ Clinical data

Methods

n/a	Involved in the study
☒	☐ ChIP-seq
☒	☐ Flow cytometry
☒	☐ MRI-based neuroimaging
