# Supplementary material for: Comparative genomics reveals the origin of fungal hyphae and multicellularity
Source: Nat Commun. 2019 Sep 9;10:4080. doi: 10.1038/s41467-019-12085-w (PMC6733946; doi:10.1038/s41467-019-12085-w)
Supplement: Supplementary file 4 — Description of Additional Supplementary Files [file 41467_2019_12085_MOESM4_ESM.pdf]

Description of Additional Supplementary Files

File Name: Supplementary Data 1
Description: List of 72 genomes/species used in the analyses

File Name: Supplementary Data 2
Description: Copy-numbers of hyphae morphogenesis gene families across 72 species

File Name: Supplementary Data 3
Description: 651 hypha morphogenesis-related genes

File Name: Supplementary Data 4
Description: Gene families whose origins mapped to BCZ nodes

File Name: Supplementary Data 5
Description: Results of the Fisher's exact test comparing the number of duplications mapped to BCZ nodes in each cluster to the genome-wide number of duplications

File Name: Supplementary Data 6
Description: Welch's t-test of basic genomic features of unicellular and multicellular fungi

File Name: Supplementary Data 7
Description: Domain architecture changes in unicellular and filamentous fungi

File Name: Supplementary Data 8
Description: List of 414 gene families identified by ANOVA

File Name: Supplementary Data 9
Description: Fisher's exact test of gene loss and gene retainment in yeast-like fungal clades

File Name: Supplementary Data 10
Description: Mcl clustering of 72 genomes

File Name: Supplementary Data 11
Description: Concatenated alignment of 72 species used for species tree inference

File Name: Supplementary Data 12
Description: RAxML species tree for 72 species

File Name: Supplementary Data 13
Description: IQTree species tree for 72 species

File Name: Supplementary Data 14
Description: PhyloBayes species tree for 72 species

File Name: Supplementary Data 15
Description: Gene trees for 362 hyphae morphogenesis gene clusters

File Name: Supplementary Data 16
Description: Gene trees for 414 newly predicted gene clusters

File Name: Supplementary Data 17
Description: Custom perl and R scripts for counting duplication rates
